# Study on the mechanism of no. 8 burn ointment in burn treatment based on network pharmacology and experimental verification

**DOI:** 10.3389/fphar.2025.1511741

**Published:** 2025-07-21

**Authors:** Le Qiu, Danlei Xing, Yuanqiang Zhu, Zhiwei Zhuang, Yexiang Sun, Peng Gong, Xulin Chen

**Affiliations:** ^1^ Department of Burns, The First Affiliated Hospital of Anhui Medical University, Hefei, China; ^2^ Department of Pharmacy, The First Affiliated Hospital of Anhui Medical University, Hefei, China

**Keywords:** No.8 burn ointment, network pharmacology, burn, HIF-1α, molecular docking

## Abstract

**Introduction:**

As a traditional Chinese compound formulation, No.8 burn ointment has demonstrated notable efficacy over half a century of clinical use, particularly in facilitating the healing of burn wounds.

**Methods:**

This study integrates clinical data, liquid chromatography-mass spectrometry, network pharmacology and experimental validation to elucidate the mechanisms by which No.8 burn ointment promotes burn wound healing and to identify its key active components.

**Results:**

The findings reveal that the ointment may accelerate wound repair by modulating the HIF-1 signaling pathway. The network pharmacology analysis has identified HIF-1α as a core target of the No.8 burn ointment’s action and has identified the key active molecules β-Elemonic acid and Rubiadin, which interact with the HIF-1α protein and influence the HIF-1 pathway.

**Conclusion:**

This research not only provides a theoretical foundation for the selection of targeted drugs in burn treatment and the optimization of traditional Chinese compounds but also provides new ideas for future research in related fields.

## 1 Introduction

Burns refer to a type of tissue injury caused by high temperatures, electric current, radiation, or corrosive chemicals, typically affecting the skin and mucous membranes, with severe cases extending to the subcutaneous and submucosal tissues (Jeschk et al., 2020). As a global public health issue, burn-related cases result in approximately 180,000 deaths annually. Statistical data indicate that the incidence is particularly high in low- and middle-income countries, especially within the regions of Africa and Southeast Asia designated by the World Health Organization, where burn cases account for about two-thirds of the total. The situation in China is also severe, according to incomplete statistics, approximately 26 million people are affected by burns each year, representing 2% of the national population, with burn-related fatalities ranking second only to traffic accidents (Monafo and West, 1990; Finnerty et al., 2016; Rowan et al., 2015).

Burn treatment research underscores the importance of integrated therapeutic strategies that are differentiated across various stages of recovery. In the early stages of treatment, the goal is to swiftly limit and control the inflammatory process induced by the trauma and to prevent potential infections (George et al., 2021). The immune response during this phase is characterized by the migration of monocytes and neutrophils to the damaged tissue, where they play a crucial role in clearing foreign bodies, necrotic tissue, and halting the spread of infection (Sierawska et al., 2022). The combined use of antibiotics and appropriate wound debridement techniques has become the standard practice for this stage (Nguyen et al., 1996). As burn wounds progress into the reparative healing phase, the focus of treatment shifts to supporting tissue regeneration and reconstruction. In this late stage, strategies encompass a range of biofactor therapies, metabolic support, and physical treatments aimed at promoting the proliferation and migration of fibroblasts and keratinocytes to the wound area. These cell types are crucial for constructing new extracellular matrix and facilitating epidermal regeneration (Rowan et al., 2015; Knoedler et al., 2023). Studies have demonstrated the effectiveness of applying growth factors such as epidermal growth factor and fibroblast growth factor to facilitate these processes (Wei et al., 2022). However, current treatment modalities still face significant challenges in controlling infection and optimizing scar healing.

No.8 burn ointment is a traditional Chinese medicine compound preparation developed by the First Affiliated Hospital of Anhui Medical University in the 1970s. It is carefully composed of traditional Chinese medicinal materials such as rheum officinale, Chinese gall, sanguisorba, borneol. After half a century of clinical practice, this preparation has demonstrated significant efficacy in treating various burn cases, gaining widespread application and recognition. Particularly in the late stages of burn healing, No. 8 burn ointment has shown excellent utility, promoting the repair of the wound and the restoration of skin function. Although the precise mechanisms of action of No.8 burn ointment remain to be fully elucidated, existing research findings suggest that the components within it possess significant bioactivity. For instance, the aloe emodin-like substances found in rheum officinale exhibit anti-inflammatory and detoxifying effects (Hu et al., 2021). The tannins present in Chinese gall in hemostasis and enhance the local tissue’s resistance to infection (Guimarães et al., 2023). Sanguisorba’s hemostatic properties and its ability to promote angiogenesis are particularly important for wound healing (Zhang et al., 2018). Borneol may assist in the treatment process by alleviating pain at the wound site and reducing local inflammatory responses (Nisar et al., 2023). The combined effects of these ingredients provide an effective traditional Chinese medicine option for the treatment of burns. Future research directions will emphasize the detailed dissection of the active components within this formulation, investigating its mechanisms of action at the cellular and molecular levels using contemporary scientific techniques. Additionally, rigorous randomized controlled trials will be conducted to further substantiate its clinical effectiveness. The goal is to optimize the therapeutic outcomes of No.8 burn ointment and broaden its potential applications in the domain of trauma treatment.

The concept of network pharmacology was first introduced by British pharmacology expert Professor Andrew L. Hopkins in 2007. This theoretical framework aims to lead an innovative path in drug design through systems biology and the analysis of multi-component networks, gradually becoming an essential strategy for advancing the development of lead compounds and novel drugs (Nogales et al., 2022). Network pharmacology particularly emphasizes adopting a systems biology perspective to comprehensively explore the complex patterns of interactions between drugs and their biological targets within multi-level biomolecular networks. This approach facilitates the discovery of drug and traditional Chinese medicine active components, as well as the systematic elucidation of their mechanisms of action (Zha et al., 2023). Additionally, the research findings in this field have demonstrated significant application value in the development of drug combination therapies, understanding the logic of traditional Chinese medicine compatibility, and elucidating the mechanisms of traditional formulas. The methods of network pharmacology offer innovative research perspectives for exploring complex TCM systems and provide a solid theoretical foundation for practical aspects such as clinical medication decision-making, drug discovery, and development (Zha et al., 2023).

The central objective of this research project is to delve into the molecular mechanisms by which No.8 burn ointment promotes the healing of burn wounds and to identify its key active components. By integrating network pharmacology with experimental validation, this study aims to provide a solid scientific foundation for uncovering the comprehensive mechanisms of No.8 burn Ointment. The research outcomes are expected to not only guide the discovery of more targeted and potent small molecule drugs but also offer essential theoretical support for the scientific modification and innovation of traditional Chinese compound formulas, thereby enhancing the overall level of clinical burn treatment.

## 2 Experimental materials and methods

### 2.1 Preparation of No.8 burn ointment

The main components of No. 8 burn ointment include: rheum officinale, Chinese gall, sanguisorba, calamine, beewax, sesame oil. Initially, 50 g each of rheum officinale, Chinese gall, sanguisorba officinalis, and calamine were thoroughly pulverized and mixed together for later use. Subsequently, 1.85 kg (2 L) of sesame oil is heated to boiling (215°C), at which point 25 g of beeswax is added and stirred uniformly to cool. Once the sesame oil reaches a state where droplets solidify into oil (approximately 100°C), the mixture of the four traditional Chinese medicines is added to the oil and rapidly stirred continuously to ensure thorough and even mixing as the mixture cools. After the sesame oil has completely cooled to the room temperature, 5 g of borneol powder is added and thoroughly stirred, yielding a suspended oily liquid. The mixture is then filtered through sterile gauze to remove impurities. Once the medicine has cooled and solidified into an ointment, 5 g is applied to a standard sterile gauze measuring 30 cm in length and 10 cm in width, creating a gauze impregnated with No.8 burn Ointment for use.

### 2.2 Prediction of potential targets for burn treatment with No.8 burn ointment

Obtain the predicted target information for the active components of Baohealing Ointment No. 8 from the SuperPred database (Gallo et al., 2022) and compare with the UniProt database to eliminate any duplicate targets (Bateman et al., 2023). Additionally, collect disease targets related to “burns” from the Genecards database (Rebhan et al., 1997), the DrugBank database (Wishart et al., 2018) and the DisGeNET database (Piñero et al., 2020). The intersection of these targets represents the core therapeutic targets of No.8 burn Ointment for the treatment of burns.

### 2.3 Construction of active compounds-disease target network

Import the core targets into the STRING database (Szklarczyk et al., 2021) to obtain a Protein-Protein Interaction network map and download the TSV format file. Set the minimum required interaction score to 0.4. Introduce the active compounds and potential targets from No.8 burn Ointment into Cytoscape 3.8.0 software to construct a compound-target-disease network. Utilize Cytoscape 3.8.0 software for the visualization of the PPI network map TSV format file obtained from the STRING database and analyze it using the Degree algorithm.

### 2.4 Enrichment analysis of potential targets

Utilize the Metascape database (Zhou et al., 2019) to perform Gene Ontology (GO) enrichment analysis and Kyoto Encyclopedia of Genes and Genomes (KEGG) enrichment analysis on the core targets. The threshold for the identification of GO and KEGG pathways is set at P < 0.05. Identify the targets within the enriched pathways through the KEGG database (Kanehisa and Goto, 2000).

### 2.5 Molecular docking based on AutoDuck

Download the chemical structures of compounds with potential pharmacological activity from No.8 burn Ointment in the PubChem database (Kim et al., 2023), and use ABGUI software to convert the structural formats. Obtain the 3D structures of targets from the Protein Data Bank (https://www.rcsb.org/). Autoduck Tools 1.5.7 Employ Autodock Tools 1.5.7 for molecular docking of the active compounds with the targets. Visualize the docking results using PyMOL software.

### 2.6 LC-MS/MS conditions

LC-MS/MS analysis was performed on a Agilent ultra-high performance liquid chromatography 1290 UPLC system with a Waters UPLC BEH C18 column (1.7 μm 2.1*100 mm). The column temperature was set at 55°C and the sample injection volume was set at 5 μL. The flow rate was set at 0.5 mL/min. The mobile phase consisted of 0.1% formic acid in water (A) and 0.1% formic acid in acetonitrile (B). The multi-step linear elution gradient program was as fo lows: 0–11 min, 85%–25% A; 11–12 min, 25%–2% A; 12–14 min, 2%–2% A; 14–14.1 min, 2%–85% A; 14.1–15 min, 85%–85% A; 15–16 min, 85%–85% A.

An Q Exactive Focus mass spectrometer coupled with an Xcalibur software was employed to obtain the MS and MS/MS data based on the IDA acquisition mode. During each acquisition cycle, the mass range was from 100 to 1,500, and the top three of every cycle were screened and the corresponding MS/MS data were further acquired. Sheath gas flow rate: 45 Arb, Aux gas flow rate: 15 Arb, Capilary temperature: 350°C, Ful ms resolution: 70,000, MS/MS resolution: 17,500, Co lision energy: 15/30/45 in NCE mode, Spray Voltage: 4.0 kV (positive) or −4.0 kV (negative).

### 2.7 Establishment of full-thickness skin burn model

Animal experiments were conducted following the guidelines approved by the Institutional Animal Care and Use Committee of Anhui Medical University (approved No. LLSC 20231268).

Initially, SD rats (6–8 weeks), were acclimatized to the laboratory conditions for 1 week. The rats were randomly assigned to three experimental groups, each comprising six animals: the control burn group, the burn group treated with No. 8 Burn Ointment, and the burn group treated with Petroleum jelly. Prior to the procedure, the rats were anesthetized using 1% sodium pentobarbital (60 mg/kg) administered intraperitoneally. According to the standardized scalding burn model in mouse (Abdull et al., 2014), the dorsal fur of rats was carefully shaved using an electric trimmer rather than depilatory cream to prevent potential skin irritation. Standardized third-degree burn wounds were created using a cylindrical brass rod with a diameter of 1.5 cm. The rod was heated in a water bath at 100°C for 15 s, and then applied vertically to the shaved dorsal skin of anesthetized mice for 15 s to induce burns. Two burn wounds, each 15 mm in diameter, were inflicted on each mouse, corresponding to approximately 10% of the total body surface area (TBSA). To facilitate accurate wound measurement and image calibration, a metal ring with an inner diameter of 2.0 cm was gently placed around the burn area before photography. This served both to highlight the burn boundary and to provide a known reference scale. Wound images were analyzed using ImageJ software (NIH, United States). The software was calibrated using the known diameter of the ring (20 mm), and wound areas were calculated in square millimeters (mm^2^) based on the calibrated images. Pain management shall be administered through subcutaneous buprenorphine (0.05 mg/kg) when evident signs of pain was observed. And infection control included daily wound cleaning with 0.9% saline. Photographs of the wound sites were taken using a digital camera at days 0, 3, 7, 14, and 21 post-burn, and the wound areas were quantified using ImageJ software for analysis.

### 2.8 Histological staining and immunohistochemical analysis

In this study, we collected the burn wound and dorsal adjacent skin tissue of rats on postoperative day 5, 10, and 15, and then the tissue samples were processed with pathological staining.

This study utilized conventional hematoxylin-eosin (HE) staining for histomorphological observation, Masson’s trichrome staining to evaluate collagen fiber deposition, and immunohistochemistry (IHC) to detect the expression and localization of HIF-1α (Bioss, bsm-62518R, 1:200). The experimental procedures included tissue fixation, paraffin embedding, sectioning, antigen retrieval (IHC), primary antibody incubation (IHC), and corresponding chromogenic reactions, with all operations performed according to standardized protocols.

**FIGURE 1:**
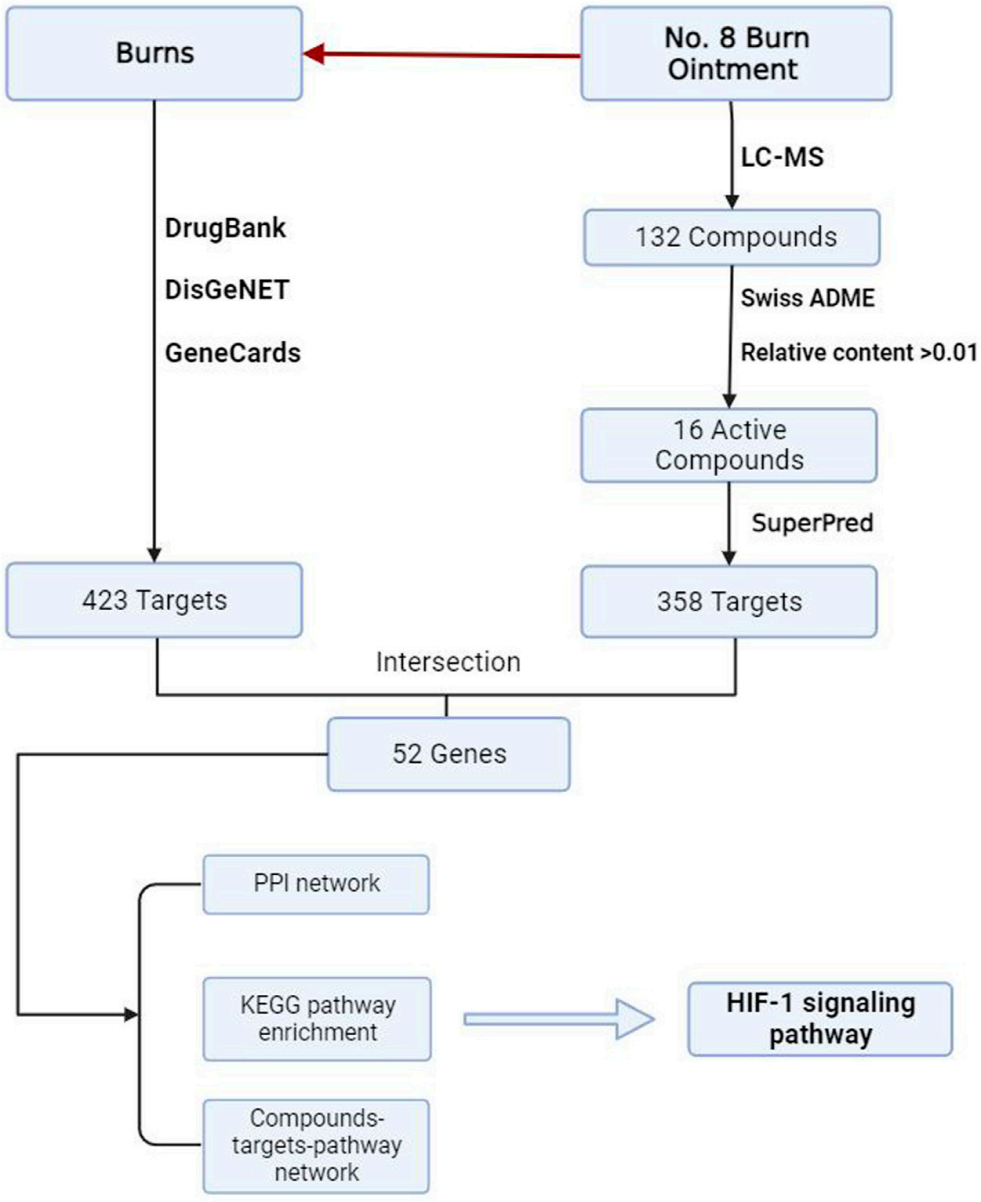
Experimental flow chart.

The experimental flow was depicted in Figure 1.

### 2.9 Clinical trial standards and treatment protocols

#### 2.9.1 Study participants

The study included 144 patients with second-degree burns who visited the outpatient clinic of the Burn and Wound Repair Department at the First Affiliated Hospital of Anhui Medical University, Gaoxin District, from October 2022 to October 2023. This research was approved by the clinical research ethics committee of the first affiliated hospital of anhui medical university, with the ethical review number 2023395.

#### 2.9.2 Inclusion criteria and exclusion criteria

The inclusion criteria were as follows: 1. Diagnosed as superficial or deep second-degree burns according to the eighth edition of the surgical textbook. 2. Presented within 24 h post-injury and confirmed as second-degree burns by at least two burn specialists. 3. Over 18 years of age. 4. No medical treatment or medication affecting wound healing 1 month prior to injury. The exclusion criteria: 1. Inpatient care or surgical intervention is required. 2. Allergic to the drug ingredients involved.

Second-degree burns typically present with blisters, significant pain, and redness, involving damage to both the epidermis and dermis layers, with varying degrees of depth.

#### 2.9.3 Treatment protocol

Patients were assigned to treatment or control groups via computer-generated random numbers. Treatment Group: Wounds were irrigated with 0.1% benzalkonium bromide solution, blisters and foreign bodies removed, and necrotic skin retained for up to 72 h. Afterwards, necrotic skin and wound secretions were removed, and the wounds were covered with No.8 burn Ointment gauze, with dressing changes either daily or every 2 days.

Control Group: Wounds were covered with Vaseline gauze after debridement and dressed as per standard care.

#### 2.9.4 Outcome measures

The average wound healing time and healing rates at 7-, 14-, 21-, and 28 days post-injury were recorded. Outcome assessors were blinded to group allocation.

Healing rate (%) = [(Initial wound area - Final wound area)/Initial wound area] * 100%. Specifically, we will clarify that the healing rate is determined by calculating the percentage reduction in wound area over the course of the study. In clinical practice, we initially measure the wound size once a week using a standard ruler, for areas where the ruler measurement is not sufficiently clear, ImageJ was used to measure the wound area at each time point. Furthermore, we will define “healed” as achieving more than 95% wound closure, based on visual inspection and the absence of any significant wound drainage or infection.

### 2.10 Statistical analyses

Data were analyzed using GraphPad Prism 8.0.2. Results are expressed as the mean ± standard deviation (SD). After the data were tested for normality, the t-test was used to determine the statistical significance between the two groups, and one-way analysis of variance was used to confirm the statistical significance among multiple groups. The levels of statistical significance are denoted as follows: *P < 0.05; **P < 0.01; ***P < 0.001.

## 3 Result

### 3.1 No.8 burn ointment promotes wound healing in burn patients

**FIGURE 2:**
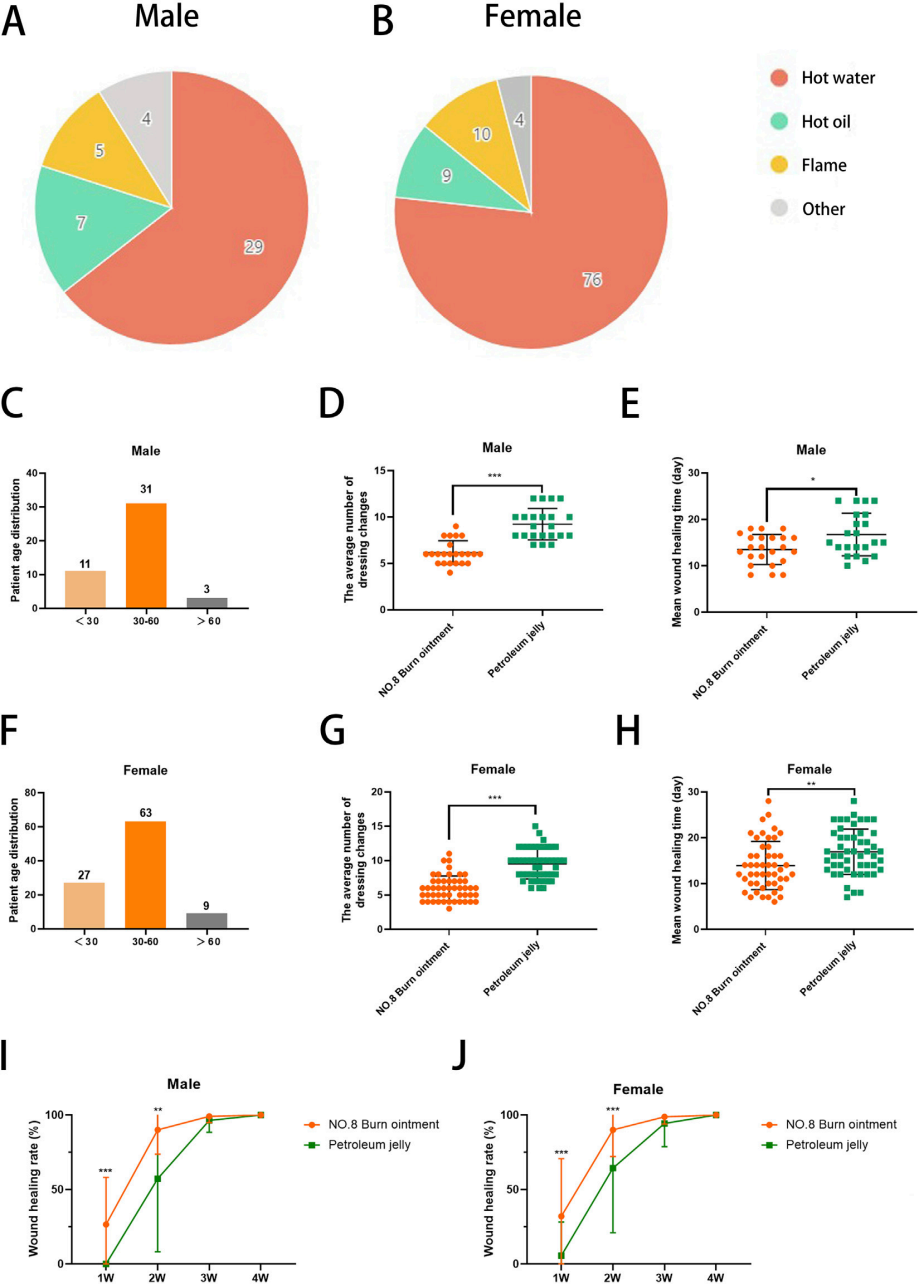
No. 8 burn ointment promotes healing of burn wounds. **(A,B)** Pie chart of burn causes. **(C)** Age distribution of male burn patients. **(D)** The average number of dressing changes in male patients. **(E)** The average wound healing time of male burn patients. **(F)** Age distribution of female burn patients. **(G)** The average number of dressing changes in female patients. **(H)** The average wound healing time of male burn patients. **(I,J)** Wound healing rate of burn patients over time.

**FIGURE 3:**
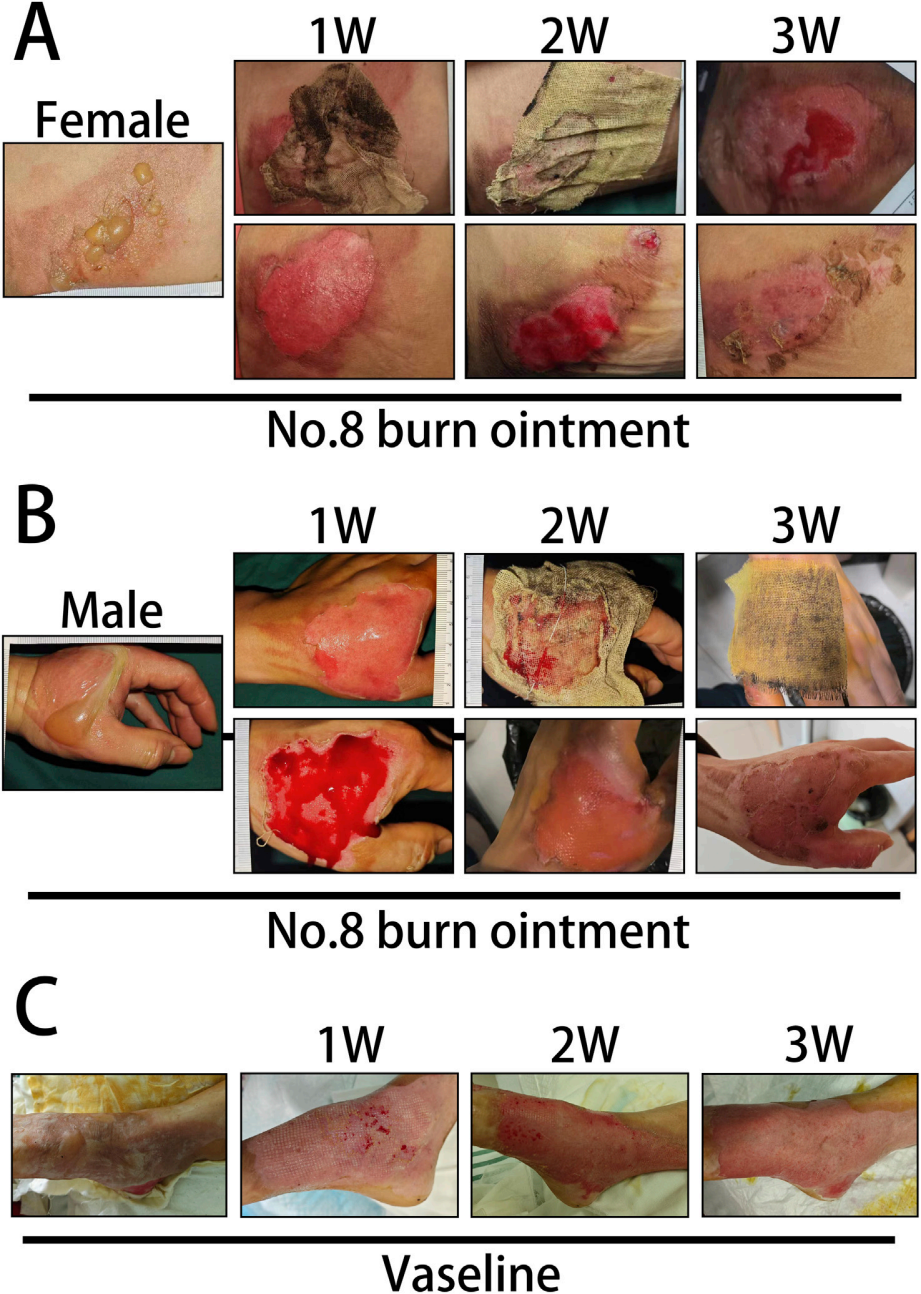
Clinical photographic records of burn patients. **(A)** Clinical photographic records after treatment with No. 8 Burn Ointment (Female patients). **(B)** Clinical photographic records after treatment with No. 8 Burn Ointment (Male patients). **(C)** Clinical photographic records after treatment with Vaseline.

In this study, we documented the etiological factors of 144 burn patients (Figures 2A,B) and presented the distribution of male and female burn patients across various age groups (Figures 2C,F), providing insight into the demographic composition of those affected by burns (Supplementary Table 1). The epidemiological data revealed that most patients were female, of working age, and hot water burns were the predominant cause. In the comparative analysis of long-term clinical treatment methods, we specifically examined the impact of two distinct therapeutic approaches, Vaseline and No.8 burn ointment on burn patients. Clinical trial results indicated that both male and female patients treated with No.8 burn ointment required significantly fewer dressing changes on average compared to those treated with Vaseline (Figures 2D,G). Additionally, the average wound healing time for both male and female patients treated with No.8 burn ointment was significantly reduced (Figures 2E,H). The wound healing time course curves vividly illustrate this result (Figures 2I,J). Furthermore, we have supplemented the clinical photographic documentation of the application of No.8 burn ointment (Figures 3A,B). Although patients treated with either Vaseline (Figure 3C) or No.8 burn ointment achieved near-complete wound healing within 3–4 weeks, the healing rate of patients treated with No.8 burn ointment was significantly higher than that of those treated with Vaseline during the first 3 weeks of therapy. In summary, the experimental results strongly suggest that No.8 burn ointment is superior to Vaseline in promoting the healing of burn wounds, significantly reducing the number of dressing changes and accelerating the wound healing process.

### 3.2 Screening of potential targets for the treatment of burns by No.8 burn ointment

**FIGURE 4:**
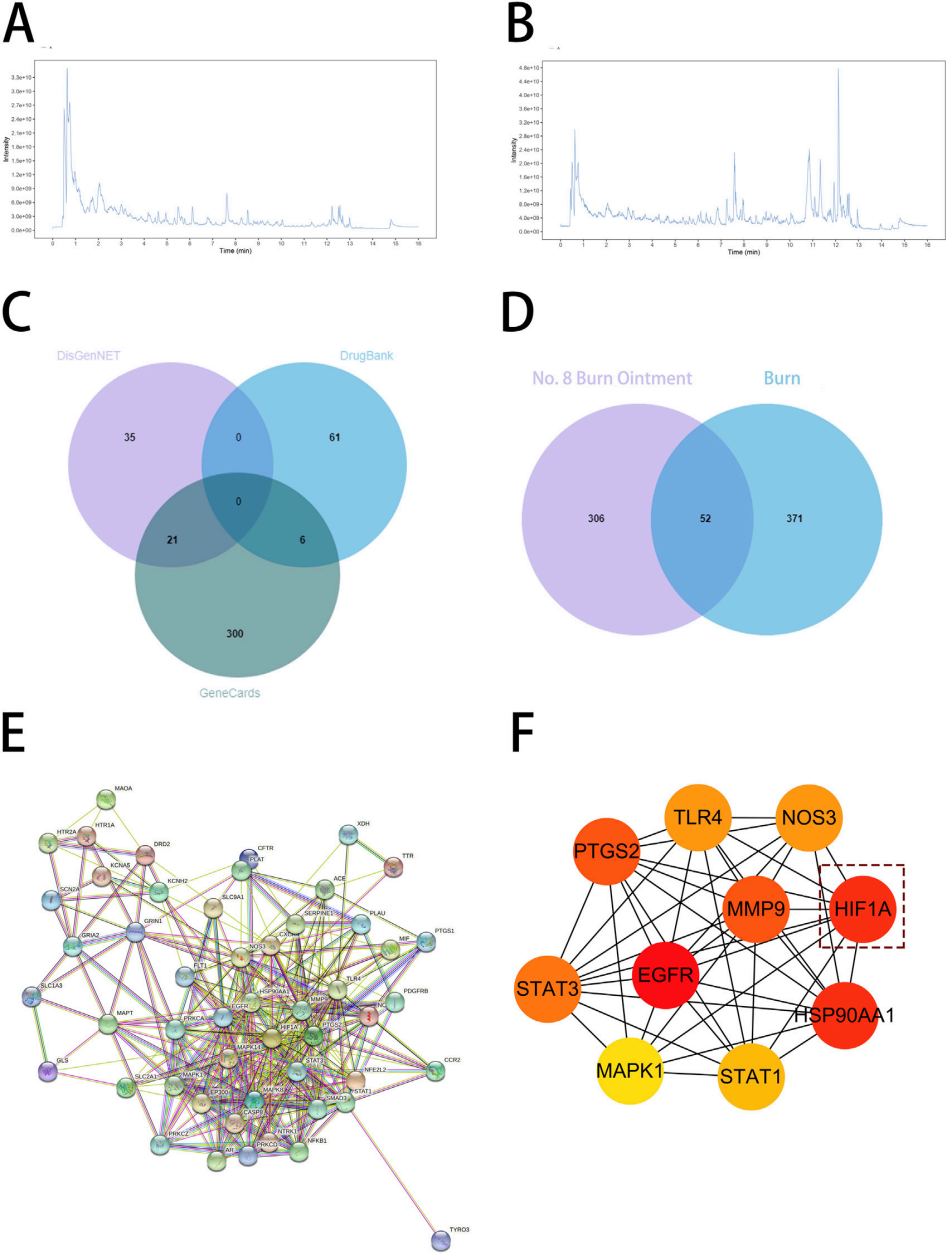
Target prediction of No. 8 burn ointment for burn treatment. **(A,B)** LC-MS/MS analysis of the constituents of No. 8 burn ointment. **(C)** Venn diagram of targets associated with burns. **(D)** Venn diagram of the intersection of No.8 burn ointment and the burn target. **(E)** Diagram of protein interaction network at intersection targets. **(F)** PPI network diagram based on Degree algorithm.

To further investigate how No.8 burn ointment facilitates the healing of burn wounds, this study utilized liquid chromatography-mass spectrometry (LC-MS) to analyze the composition of the No.8 burn ointment. By employing the McCloud online database and mzVault traditional Chinese medicine natural product database for feature peak identification, we identified 16 components present at concentrations above 1%, with genistein, syringic acid, rhein, and ellagic acid being the predominant constituents (accounting for over 52%) (Figures 4A,B). Utilizing the SuperPred database, we obtained predicted target information for 16 active components of No.8 burn ointment, which yielded 358 potential targets. Subsequently, we collected 423 disease-related targets for “burns” from three disease target databases: Genecards, DrugBank, and DisGeNET. The intersection of these targets allowed us to identify 52 core targets for the treatment of burns with No.8 burn ointment (Figures 4C,D). Additionally, we constructed a Protein-Protein Interaction network for the aforementioned 52 core targets and further analyzed it based on the Degree algorithm (Figures 4E,F). The experimental results suggest that HIF1A, EGFR, and HSP90AA1 may play significant roles in the wound healing effects of No.8 burn ointment.

### 3.3 Enrichment analysis of core targets

**FIGURE 5:**
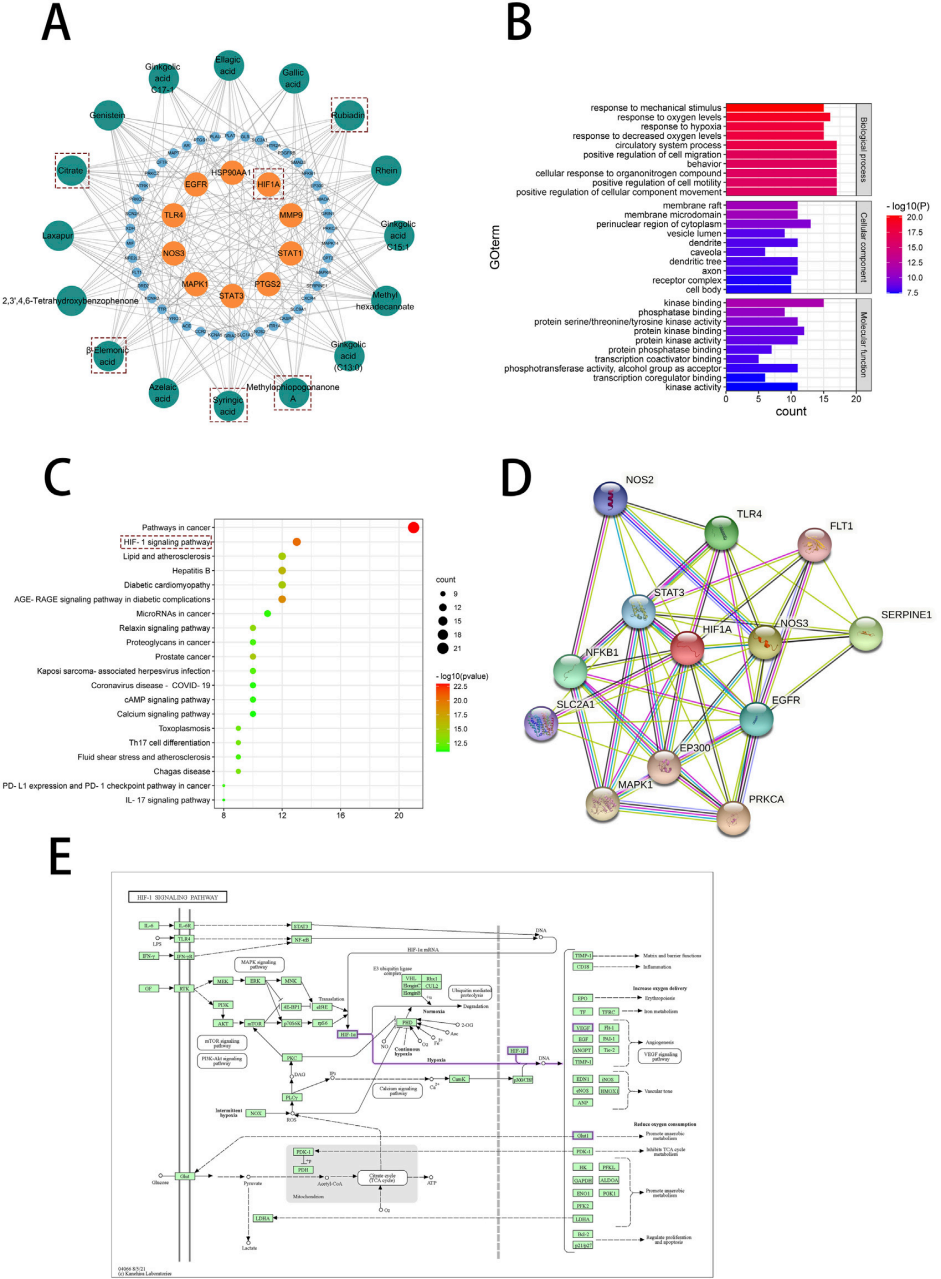
Network pharmacological analysis of No. 8 burn ointment in the treatment of burn. **(A)** Composition - target network diagram construction. **(B)** GO enrichment analysis of intersection targets. **(C)** KEGG enrichment analysis of intersection targets. **(D)** PPI network diagram. **(E)** HIF-1 signaling pathway diagram in KEGG database.

Through enrichment analysis of core targets, we sought to elucidate the biological functions and associated pathways of the gene clusters, aiming to identify potential therapeutic targets for drug intervention in disease pathogenesis. To further elaborate the relationship between the active components in No.8 burn ointment and the core targets, we constructed a compound-target network graph, highlighting the top ten targets identified by the Degree algorithm in yellow (Figure 5A). Further, GO enrichment analysis showed that the biological processes were highly enriched in “response to oxygen levels”, “response to hypoxia”, and “response to decreased oxygen levels” (Figure 5B). The experimental results of KEGG pathway enrichment also suggest that it is significantly enriched in the HIF-1 signaling pathway (Figure 5C). We constructed a Protein-Protein Interaction network for the core targets enriched in the HIF-1 signaling pathway, and the results indicated that HIF1A played a central role within this network (Figure 5D). Additionally, we delineated the downstream pathways mediated by HIF1A in the HIF-1 signaling pathway map sourced from the KEGG database (Figure 5E). The aforementioned results suggest that the therapeutic effect of No.8 burn ointment on burn wound healing may be based on its regulation of the HIF-1 signaling pathway.

### 3.4 The docking of the active small molecules of No.8 burn ointment with the molecules of the core target

**FIGURE 6:**
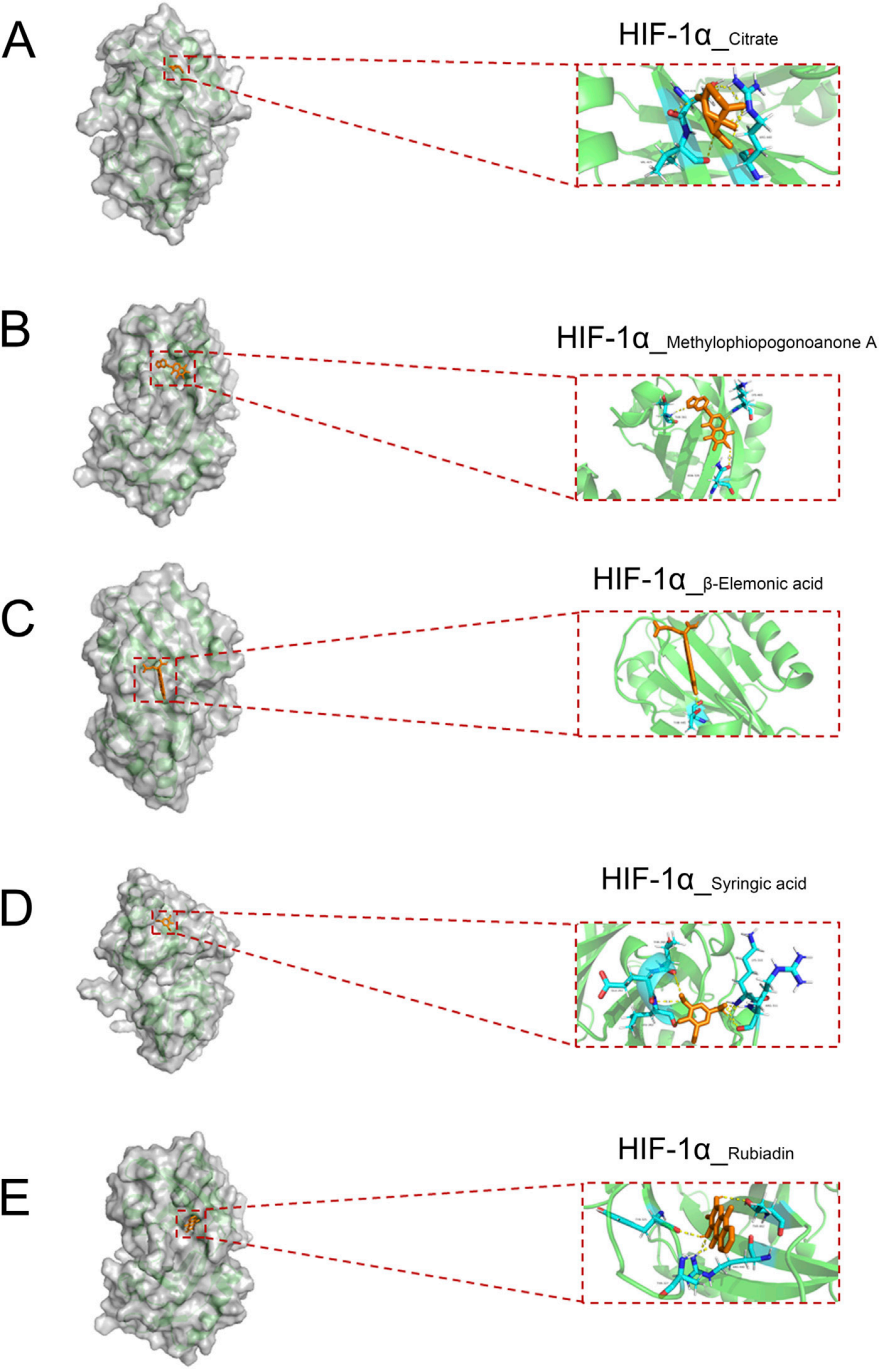
The docking of the active small molecules in No. 8 burn ointment with the core target HIF-1α. **(A)** Molecular docking results of HIF-1α and Citrate. **(B)** Molecular docking results of HIF-1α and Methylphiopogonanone A. **(C)** Molecular docking results of HIF-1α and β-Elemonic acid. **(D)** Molecular docking results of HIF-1α and Syringic acid. **(E)** Molecular docking results of HIF-1α and Rubiadin.

**FIGURE 7:**
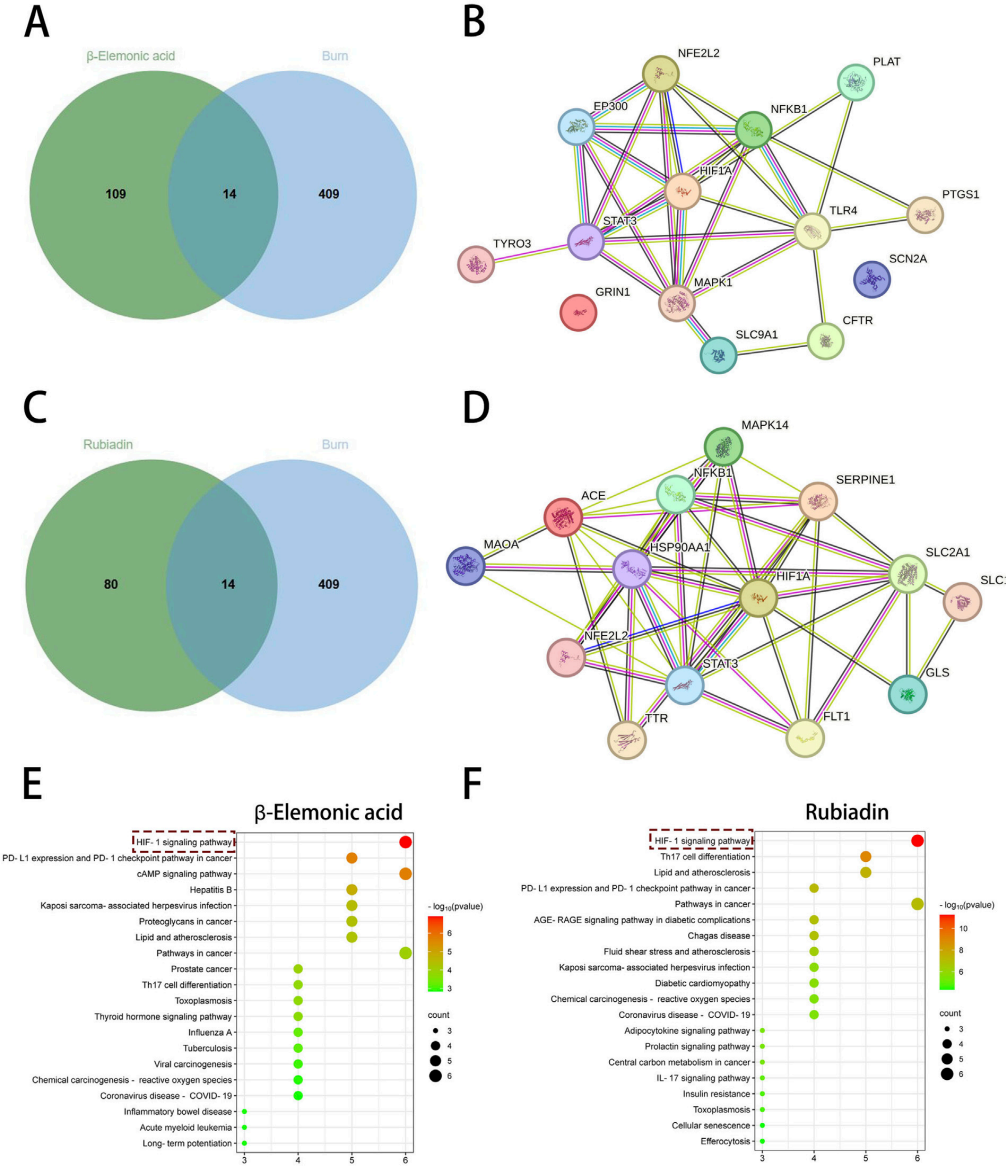
Network pharmacological analysis of β-Elemonic acid and Rubiadin in the treatment of burn. **(A)** Venn diagram of the intersection of β-Elemonic acid and the burn target. **(B)** PPI network diagram. **(C)** Venn diagram of the intersection of Rubiadin and the burn target. **(D)** PPI network diagram. **(E)** KEGG enrichment analysis of intersection targets. **(F)** KEGG enrichment analysis of intersection targets.

To further identify the active small molecules within No.8 burn ointment, we employed molecular docking to validate the binding modes of these molecules with the core target HIF-1α. The experimental results showed that Citrate formed seven hydrogen bonds with SER424, VAL425 and ARG440 in HIF-1α (Figure 6A). Methylphiopogonanone A formed three hydrogen bonds with LYS465, THR361 and ASN326 in HIF-1α (Figure 6B). β-Elemonic acid formed one hydrogen bond with THR445 in HIF-1α (Figure 6C). Syringic acid formed six hydrogen bonds with GLU261, LEU262, THR260, ARG311 and LYS310 in HIF-1α (Figure 6D). Rubiadin formed four hydrogen bonds with THR462, TYR325 and THR327 in HIF-1α (Figure 6E). We calculated the binding energy to assess the degree of complementarity between the active small molecules and HIF-1α. A lower binding energy indicates greater stability. The binding energies of HIF-1α with Citrate, Methylphiopogonanone A, β-Elemonic acid, Syringic acid, and Rubiadin were determined to be −5.57, −5.59, −8.21, −3.34, and −7.27 kJ/mol, respectively. The results indicate that β-Elemonic acid and Rubiadin bind effectively to the active site of the core target HIF-1α, suggesting that they may be key active small molecules in regulating the HIF-1 signaling pathway. Additionally, we constructed Venn diagrams for the Eleonic and Rubiadin targets with burn targets (Figures 7A,C), and for the Intersection targets, we built PPI network diagrams and conducted KEGG enrichment analysis (Figures 7B,D–F). Experimental results further confirmed the above insight, showing that the intersection targets of β-Elemonic acid and Rubiadi targets and burn targets are significantly enriched into the HIF-1 signaling pathway.

We have further evaluated the ADMET properties and physicochemical characteristics of Rubiadin and β-Elemonic acid to comprehensively assess their potential as topical dermatological agents (Supplementary Figure S1, Supplementary Table 1). As two promising candidate molecules for external application, these compounds exhibit complementary key properties. Rubiadin demonstrates ideal drug-like features: its moderate molecular weight and significant structural rigidity contribute to stable local action on the skin. The balanced number of hydrogen bond donors and acceptors ensures effective interaction with the stratum corneum while avoiding excessive hydrophilicity. Notably, this compound shows excellent skin retention properties, though its relatively low water solubility may require formulation optimization. Special attention should be paid to its skin sensitization potential, which warrants thorough safety evaluation in subsequent development. In contrast, β-Elemonic acid exhibits superior performance in several critical aspects: its exceptional metabolic stability and remarkable natural product likeness provide unique advantages. Particularly noteworthy is its extremely low systemic absorption risk, making it an ideal candidate for topical application. However, the relatively large molecular size and higher lipophilicity might affect transdermal efficiency, which would need to be addressed through advanced delivery technologies (such as nanocarriers or penetration enhancers). Taken together, Rubiadin, with its well-balanced molecular properties and good local retention, appears more suitable for developing short-term, high-concentration topical formulations. Meanwhile, β-Elemonic acid, owing to its outstanding metabolic stability and minimal systemic exposure risk, is better suited for developing sustained-release topical preparations.

### 3.5 *In vivo* evaluation of wound healing

**FIGURE 8:**
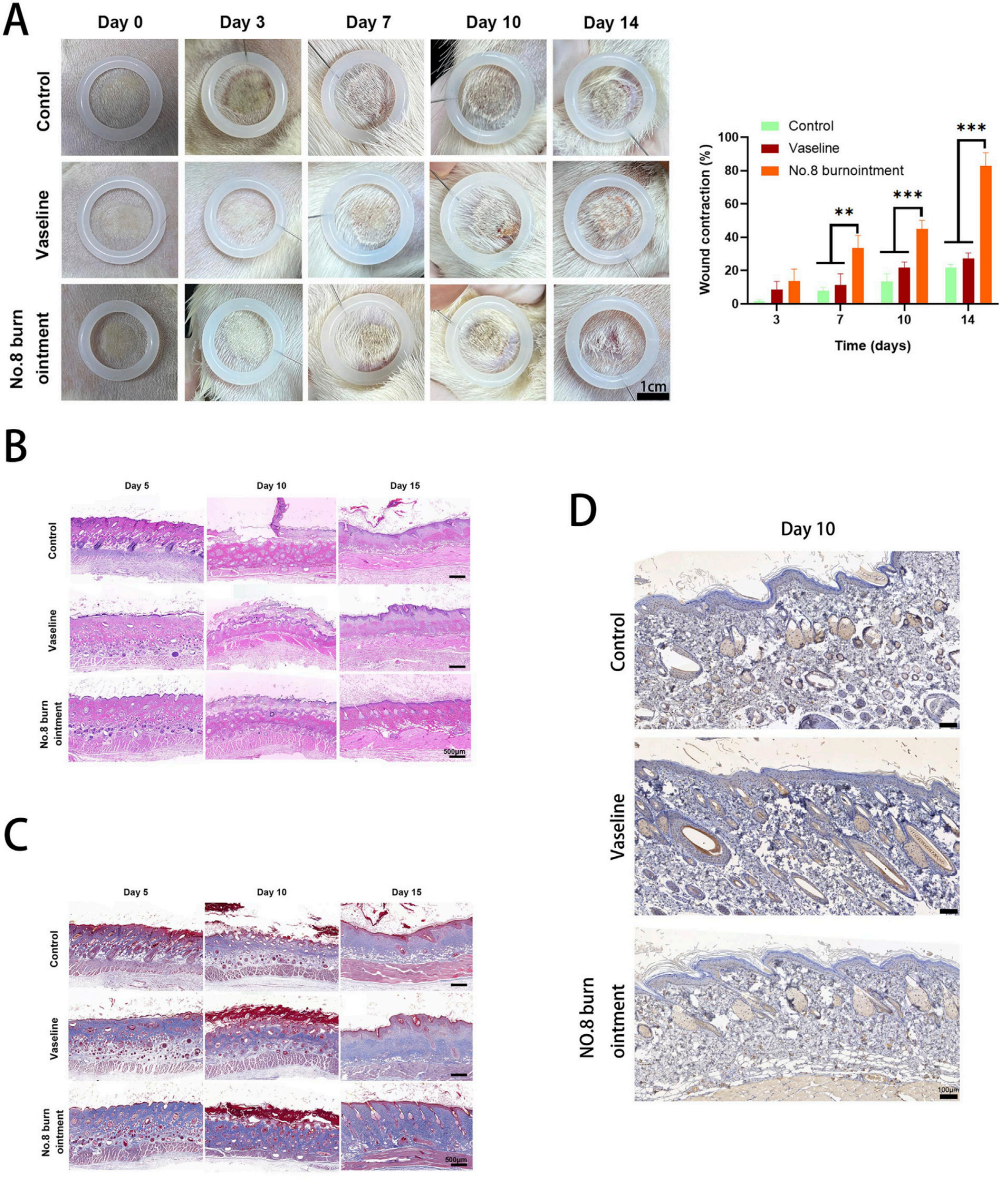
No. 8 burn ointment can promote the healing of burn wounds in rats. **(A)** Photographs of wounds treated with the different materials on days 0, 3, 7, 10, and 14. **(B)** Hematoxylin-eosin staining images of wound tissues treated with the control, Vaseline, and No.8 burn ointment. **(C)** Masson staining images of wound tissues treated with the control, Vaseline, and No. 8 burn ointment. **(D)** Immunohistochemical staining of HIF-1α in wound tissue treated with control, Vaseline, and No. 8 burn ointment.

As depicted in Figure 8A, infections were observed in all groups by day 3, with a noticeable trend towards wound healing in each group. Notably, the No.8 burn ointment group exhibited the fastest wound healing speed among all groups. By day 3, new tissue growth was observed in both the Vaseline and No.8 burn ointment groups, while less new tissue formation was observed in the blank control group. By day 7, the No.8 burn ointment group scab faster than the other two groups significantly. By the end of day 14, the newly formed skin in the No.8 burn ointment group completely covered the original wound, achieving a remarkable 100% wound healing. These findings demonstrate that the No.8 burn ointment group can promote wound healing to a certain extent, with their combined application further enhancing their ability to promote wound healing.

Wound healing is a multifaceted physiological process that necessitates assessing both the external recovery of the wound and the evaluation of inflammatory reactions and tissue regeneration within the wound. Hematoxylin-eosin staining (H&E) images of wound tissues on days 5, 10, and 15 are shown in Figure 8B. As depicted in Figure 7B, the control group exhibited considerable neutrophil infiltration on day 5, suggesting persistent infection and exacerbation of inflammation. By day 10, the No.8 burn ointment-treated wounds displayed reduced inflammatory cell infiltration compared to the Vaseline and control groups. Conversely, the control group exhibited a higher presence of inflammatory cells, suggesting that the No.8 burn ointment could mitigate inflammation during the initial stages of wound healing. Interestingly, the crust forms over the skin surface were notably present at the wound site in the No.8 burn ointment and Vaseline by day 10, whereas these appendages were scarce or absent in other groups. Furthermore, the No.8 burn ointment group exhibited a higher proportion of extracellular matrix, indicating fast granulation tissue growth by day 15.

Collagen formation plays a pivotal role in the wound healing process by promoting cell migration and serving as the foundation for extracellular matrix deposition during wound healing and proliferation. Masson staining was employed to assess collagen deposition and arrangement in the new wound tissue (Figure 8C). The No.8 burn ointment group exhibited clear presence of skin appendages such as hair follicles and sweat glands at the wound site on day 15, and well-organized collagen accumulation, whereas these skin appendages were rare or absent in the Vaseline and control group, respectively. Furthermore, the No.8 burn ointment group with the scar epithelium thickness being uniform and similar to the surrounding skin, and the skin became flatter. In comparison, collagen staining in the No.8 burn ointment group was similar to the peripheral undamaged subcutaneous tissue with the orderly collagen fibers accumulated structure. These observations suggest that the treatment with No. 8 Burn Ointment has a positive impact on wound healing, promoting the orderly deposition of collagen and the regeneration of skin appendages. Furthermore, on the tenth day following burn wound healing, we conducted an immunohistochemical staining analysis for HIF-1α, which revealed a significantly elevated expression level in the group treated with No. 8 Burn Ointment (Figure 8D). This finding further validates our earlier hypothesis that No. 8 Burn Ointment may enhance the reparative capacity of burn wounds by activating the HIF-1 signaling pathway, thereby promoting collagen synthesis and facilitating the smooth progression of the wound healing process.

## 4 Discussion

Burns are a common form of trauma worldwide, inflicting not only severe pain, potential life-threatening complications, and long-term functional impairments on patients, but also imposing a significant burden on healthcare systems. The cornerstone of effective burn treatment lies in the acceleration of wound healing to mitigate the risks of infection, scar formation, and loss of function (Rowan et al., 2015; Snell et al., 2013). However, due to the complex physiological environment of burn wounds and numerous influencing factors, including cellular necrosis, inflammatory responses, tissue ischemia, and immune dysfunction, there remains a scarcity of highly effective and targeted therapeutic approaches. No.8 burn ointment is a compound traditional Chinese medicine formulation developed by the First Affiliated Hospital of Anhui Medical University in the 1970s. At that time, experts created twelve distinct burn ointments, among which the eighth formulation, through half a century of clinical validation, has consistently demonstrated favorable therapeutic outcomes for burns of various etiologies. While its efficacy has been demonstrated through practical application, the specific mechanisms underlying its therapeutic effects remain unclear, which limits its integration into modern medical practices and underscores the urgent need for in-depth investigation into its mechanisms of action. This study aims to investigate No.8 burn ointment as the subject of research, integrating extensive clinical data with network pharmacology to systematically elucidate its molecular and cellular mechanisms in promoting the healing of burn wounds. Our focus is on the potential regulatory effects of No.8 burn ointment on the HIF-1 signaling pathway, which plays an essential role in the response to hypoxia and the wound healing process. Research has shown that this pathway has a central role in promoting tissue repair during disease state. Through a multi-level, multi-angle investigation ranging from the molecular to the systemic, this project aspires to provide comprehensive scientific evidence for the mechanisms of action of No.8 burn ointment and to offer novel strategies and theoretical support for the advancement of modern burn treatment.

In this study, we provide evidence supporting the efficacy of No.8 burn ointment in promoting wound healing in burn patients, based on clinical data from 144 cases and extensive clinical practice. We employed liquid chromatography-mass spectrometry (LC-MS) and network pharmacology analysis to identify HIF1α as a core target associated with the promotion of burn wound healing by the ointment. Additionally, we identified key active small molecules that interact with the HIF-1α protein and influence the HIF-1 signaling pathway. This discovery was confirmed through molecular docking experiments and further validated in a mouse burn model.

Traditional Chinese medicine compound formulations have been empirically validated for their safety and efficacy through clinical practice. However, due to their complex composition and intricate therapeutic mechanisms, we aim through this preliminary study to provide valuable directions for further in-depth exploration. While the outcomes of this study are indeed encouraging, we acknowledge certain limitations inherent in our approach. Firstly, the size of burn model and clinical samples are relatively small and lacks diversity, which may restrict the generalizability of our findings to broader populations. Secondly, our research has primarily concentrated on the principal active components of No.8 burn ointment, potentially overlooking the contributions of secondary constituents or synergistic effects. To bolster the reliability of our research and to further explore the therapeutic potential of the ointment, future studies should encompass a broader patient demographic and conduct multicenter randomized controlled trials. A deeper investigation into the other active components within the No.8 burn ointment will aid in a more comprehensive understanding of its molecular mechanisms and potential synergistic actions. Functional studies on HIF-1α and other target genes will clarify their specific roles in the wound healing process. The refinement of traditional Chinese medicine components and the selection of active molecules are crucial for the standardization and improvement of the drug, significantly impacting the development of burn treatment medications. The current research is relatively preliminary, and we shall further investigate the specific molecular mechanisms of the relevant signaling pathways in subsequent studies. Overall, our research provides valuable evidence for the integration of traditional Chinese medicine into modern medical practice, yet future studies must overcome current limitations and challenges to enhance the contribution of traditional Chinese medicine in the field of burn treatment.

## 5 Conclusion

In summary, by integrating clinical data, network pharmacology analysis, and animal experiments, this study has discovered that No.8 burn ointment can promote the healing of burn wounds by regulating the HIF-1 signaling pathway. It is preliminarily hypothesized that the mechanism by which it regulates the HIF-1 pathway may involve the active small molecule components β-Elemonic acid and Rubiadin. This research offers a new perspective for the selection of active small molecules targeted for burn treatment and provides a theoretical basis for the subsequent modification of traditional Chinese compound formulas. However, further clinical studies and in-depth mechanistic investigations are necessary to confirm and optimize this finding, thereby advancing the field of burn treatment. The predictive results of this study based on network pharmacology require further experimental validation to confirm their biological significance. Overall, this study provides significant insights into the mechanism by which No.8 burn ointment facilitates burn wound healing, laying the groundwork for targeted drug selection in burn treatment and the improvement of traditional Chinese compound formulas. The findings of this research hold promise for offering more effective and safe treatment options for burn patients and charting new directions and ideas for further research in the field.

## Data Availability

The original contributions presented in the study are included in the article/Supplementary Material, further inquiries can be directed to the corresponding author.

## Supplementary material

SUPPLEMENTARY FIGURE S1 Radar Chart of Physicochemical Properties of β-Elemonic acid and Rubiadin. MV(Molecular Weight); nRig (Number of rigid bonds); fChar (Formal charge); nHet (Number of heteroatoms); MaxRing (Number of atoms in the biggest ring); nRing (Number of rings); nRot (Number of rotatable bonds); TPSA (Topological Polar Surface Area); nHD (Number of hydrogen bond donors); nHA (Number of hydrogen bond acceptors); logD (The Logarithm of the n-octanol/water distrbution coefficient at pH = 7.4); logS (The Logarithm of Aqueous Solubility Value); logP (The Logarithm of the n-octanol/water distrbution coefficients).
