# Supplementary figures and images for: Study on the mechanism of no. 8 burn ointment in burn treatment based on network pharmacology and experimental verification
Source: Front Pharmacol. 2025 Jul 21;16:1511741. doi: 10.3389/fphar.2025.1511741 (PMC12319001; doi:10.3389/fphar.2025.1511741)

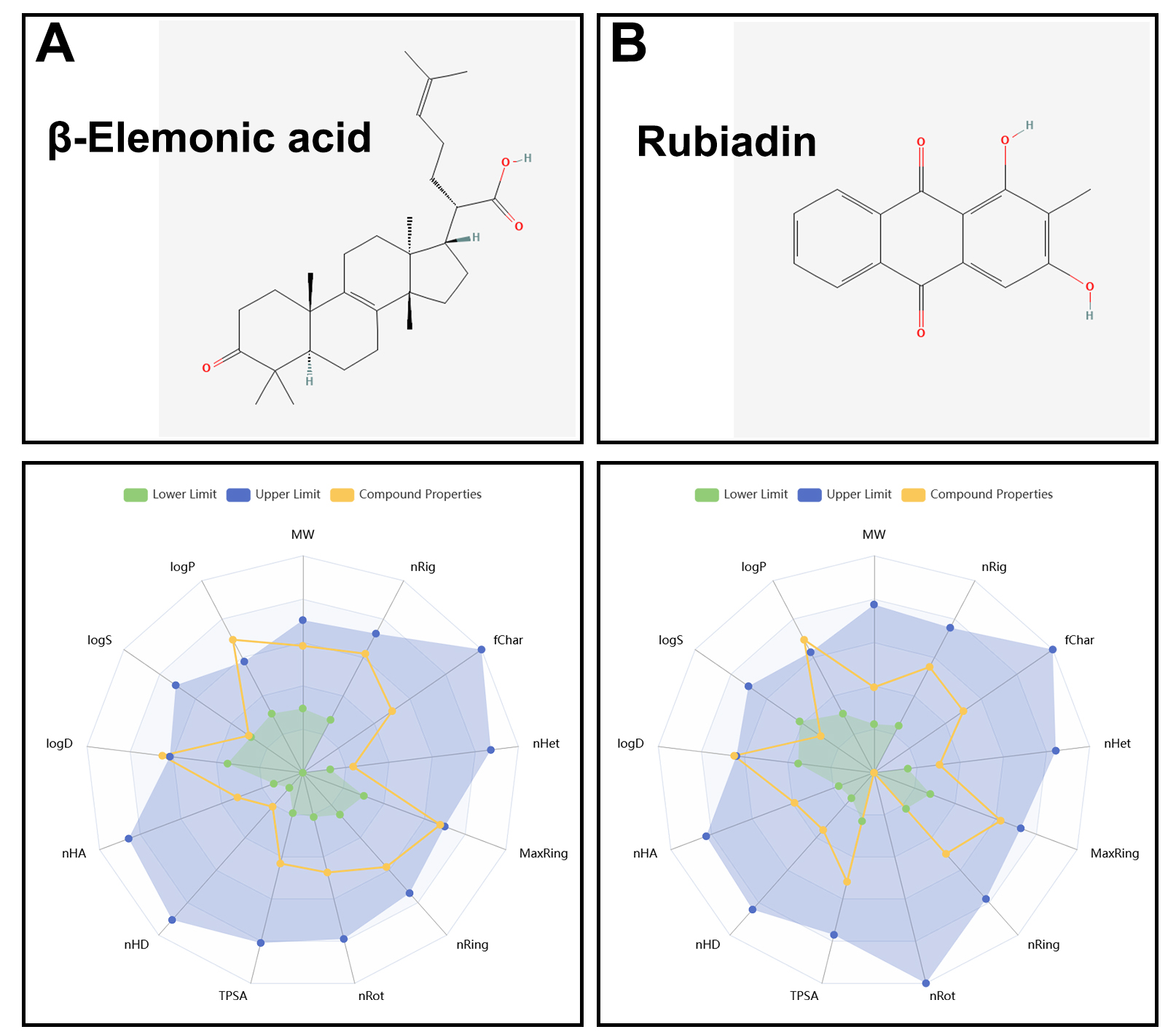

Supplement: Supplementary file 1 [file Image1.jpg]
